# A Sweet Philosophy

**Published:** 1878-08

**Authors:** 


**A Sweet Philosophy.**—The celebrated teacher, Rabbi Meir, sat during the whole of one Sabbath-day in the public school, instructing the people. During his absence from the house, his two sons died—both of them of uncommon beauty, and enlightened in the law. His wife bore them to her bed chamber, laid them upon the marriage-bed, and spread a white covering over their bodies. In the evening the Rabbi came home.

She reached him a goblet. He praised the Lord at the going out of the Sabbath, drank, and asked:

"Where are my two sons, that I may give them my blessing? I repeatedly looked round the school, and I did not see them there. Where are my sons, that they, too, may drink of the cup of blessing?"

"They will not be far off," she said, and placed food before him, that he might eat.

He was in a gladsome and genial mood; and when he had said grace after the meal, she thus addressed him:

"Rabbi, with thy permission I would fain propose to thee one question."

"Ask it, then, my love," he replied.

"A few days ago a person intrusted some jewels to my custody; now he demands them again. Should I give them up?"

"This is a question," said Rabbi Meir, "which my wife should not have thought it necessary to ask. What! wouldst thou hesitate or be reluctant to restore to every one his own?"

"No," she replied; "but yet I thought it best not to restore them without acquainting thee therewith."

She then led him to the chamber, and stepping to the bed, took the white covering from the dead bodies.

"Ah! my sons, my sons!" loudly lamented the father. "My sons, the light of my eyes, and the light of my understanding! I was your father, but you were my teachers in the law."

The mother turned away and wept bitterly. At length she took her husband by the hand and said: "Rabbi, didst thou not teach me that we must not be reluctant to restore that which was intrusted to our keeping? See, the Lord gave, and the Lord has taken away, and blessed be the name of the Lord."

"Blessed be the name of the Lord!" echoed Rabbi Meir; "and blessed be his name for thy sake, too; for well it is written: 'Whoso hath found a virtuous wife hath a greater treasure than costly pearls. She openeth her mouth with wisdom, and in her tongue is the law of kindness.'"

